# Modeling *In Vitro* Fertilization Data Considering Multiple Outcomes Observed among Iranian Infertile Women

**DOI:** 10.22074/ijfs.2018.5187

**Published:** 2018-01-05

**Authors:** Azadeh Ghaheri, Aliakbar Rasekhi, Reza Omani Samani, Ebrahim Hajizadeh

**Affiliations:** 1 Department of Biostatistics, Faculty of Medical Sciences, Tarbiat Modares University, Tehran, Iran; 2 Department of Epidemiology and Reproductive Health, Reproductive Epidemiology Research Center, Royan Institute for Reproductive Biomedicine, ACECR, Tehran, Iran

**Keywords:** Cluster Analysis, Infertility, *In Vitro* Fertilization

## Abstract

**Background:**

Women undergoing *in vitro* fertilization (IVF) cycles should successfully go through multiple points during the procedure (i.e., implantation, clinical pregnancy, no spontaneous abortion and delivery) to achieve live births. In this study, data from multiple cycles and multiple points during the IVF cycle are collected for each individual to model the effects of factors associated with success at different stages of IVF cycles in Iranian infertile women.

**Materials and Methods:**

This historical cohort study includes 996 assisted reproductive technology (ART) cycles of 511 infertile women. Covariates considered in this study were women’s age, type of cycle (fresh or frozen embryo transfer), number of embryos transferred and having polycystic ovarian syndrome during IVF cycles. Generalized estimating equations were used for calculation of odds ratio (OR) and 95% confidence intervals (95% CI) of success at different stages during IVF cycles. Cluster-weighted generalized estimating equations (CWGEE) was also fitted to handle informative cluster size.

**Results:**

After adjusting for potential confounders, it was seen that receiving frozen embryo transfer was associated with higher odds of success compared to receiving fresh embryo transfer (adj OR: 2.26, 95% CI: 1.66-3.07); however, cycles with fresh embryo transfer exhibited better results in clinical pregnancy compared to those receiving frozen embryo. Being in the age category of 38 to 40 was associated with lower odds of success compared to the reference category (<35) in CWGEE model (adj OR: 0.67, 95% CI: 0.45-1.00). The number of embryos transferred was positively associated with the odds of success in CWGEE (adj OR: 1.21, 95% CI: 1.03-1.42) as well as the GEE model.

**Conclusion:**

Receiving frozen embryo was positively associated with odds of success compared to cycles with fresh embryo. The number of embryos transferred and women’s age were significantly associated with odds of success.

## Introduction

In Iran, the average rate of infertility, primary and secondary infertility and current infertility is estimated to be 10.9% [95% confidence intervals (CI): 7.4-14.4], 10.6% (95% CI: 5.3-16.0), 2.7% (95% CI: 1.9-3.5) and 3.3% (95% CI: 2.7-3.8), respectively (1). Currently, assisted reproductive technology (ART) is increasingly used as a widely accepted treatment for infertile couples (2). The increase in popularity of ART, the factor influencing its outcome and the importance of success rate have motivated researchers towards modeling ART success rates and identifying factors that affect it in different ways (3-5).

An *in vitro* fertilization (IVF) process involves retrieving eggs (oocytes) and sperm from female and male, respectively and allowing sperm to fertilize the eggs; the resulting embryo(s) are then transferred to the uterus and hormones are administrated to aid embryo implantation (6). Women undergoing IVF should go successfully through multiple points during the procedure (i.e., chemical pregnancy, clinical pregnancy, having no spontaneous abortion (SAB) and a successful delivery) to achieve live births; therefore, in IVF data, success probabilities at each stage are conditional on success at the previous stage. Furthermore, pregnancy outcomes are believed to be correlated within different cycles of a woman and women’s reproductive outcomes in previous ART cycles are believed to influence the outcomes of their current cycle; so, there is a need to consider previous cycles data rather than simply considering those of the current cycle.

Most studies on ART data have only inspected a part of infertile women’s data (7-10). Multiple types of IVF failure and multiple IVF cycles experienced by each woman, have not simultaneously been considered in previous studies. Maity et al. (11) presented an approach based on ideas of discrete survival analysis of IVF data with multiple cycles and multiple failure types for each individual. Generalized estimating equations (GEE), which consider the correlation within clusters, can be used to fit the model presented in their study. In case of ART data, the cluster would be the woman and the cycles each woman undergoing the procedure would be the observation (subunit) within the cluster.

In the GEE analysis it is assumed that, the response is independent from the number of observations in the cluster (the cluster size) (12). However, in IVF data, the number of cycles that an infertile woman undergoes is believed to be associated with the success/failure of IVF outcome (known as informative cluster size). The model presented by Maity et al. (11) does not consider informative cluster size. In the present study, a cluster-weighted GEE (CWGEE) was used to model the factors associated with binary outcome of success/failure at different stages during IVF cycles while handling informative cluster size. The results were then compared with those of GEE model.

## Materials and Methods

This historical cohort study includes 996 cycles of 511 infertile women who were enrolled in ART treatments between April 2011 and March 2012 in Royan institute, Iran. Only women who experienced embryo transfer were eligible to be included in the present analysis. All variables in this study were defined based data extracted from the medical record of the individuals, by trained nurses. The outcome variable was success or failure at four stages: i. Chemical pregnancy [a transient increase in serum beta-human chorionic gonadotropin (ß-hCG)], ii. Clinical pregnancy (presence of an intrauterine gestational sac), iii. Spontaneous abortion (pregnancy loss before 20 completed weeks of pregnancy), and iv. Delivery (live birth of at least one baby).

Cycles resulted in failure types other than the four above-mentioned ones, were excluded from the study and couples who required donation or gestational carrier, were not eligible for enrollment. Covariates considered in this study were women’s age (under 35, 35 to 37, 38 to 40, above 40), type of cycle (fresh or frozen embryo transfer), the number of embryos transferred and having polycystic ovarian syndrome (PCOS) during IVF cycles. Some other measured covariates were woman-specific, such as age at the first cycle while some others were cycle-specific, such as type of cycle or the number of embryos transferred.

The study was approved by the Ethics board of research of Royan institute (Ethical code: EC/90/1086). Informed consent was obtained from all subjects when they intended to start the treatment. Subjects were assured that the results would be published following statistical evaluations and no personal data would be disclosed.

### Statistical analysis

The outcome at each stage (chemical pregnancy, clinical pregnancy, spontaneous abortion (SAB) and delivery) was considered as the binary response variable representing the success or failure of the stage. The probability of success occurrence at a specific stage of ART cycle, could be associated with the stage, cycle number, and covariates of interest. The main challenge is considering the correlations among repeated cycles of each woman, as well as correlations among the outcomes of multiple stages within each cycle. To consider these correlations, GEEs were used according to the model presented by Maity et al. (11), to assess the influence of covariates (women’s age, type of cycle, number of embryos transferred and having PCOS) on the binary outcomes and calculation of odds ratio (OR) and 95% CI. In usual GEE analysis, it is assumed that the outcome is independent of the number of observations in each cluster. However, concerning IVF data, the cluster size is believed to be informative or non-ignorable. In this study, a CWGEE was also fitted to handle informative cluster size. Stata software, version 13 (Stata Corp, College Station, TX, USA) was used for statistical analyses.

## Results

This study includes 511 women with a total of 996 IVF cycles, each woman having 1-3 cycles leading to embryo transfer. The mean (SD) age of women was 35.75 (5.12) years old and 86 (16.8%) of women had PCOS. Among the cycles included in this study, 585 (59%) were cycles with fresh embryo transfer and the median (inter quartile range) of the number of embryos ready for transfer was 3 (2-3).

Since the number of cycles that each woman experienced is reversely associated with the success/failure at different stages, conditional on other predictors (OR: 0.68, 95% CI: 0.52-0.89, P=0.005), cluster size is believed to be informative and CWGEE has been suggested for handling this situation (13).

GEE and CWGEE models used in this study incorporated the data from repeated IVF cycles and multiple stages, with a separate intercepts for stage (Table 1). According to this table, age was associated with odds of success in CWGEE model as women of 38-40 years old were less likely to have successful IVF outcome than women under 35 years old. However, this association was not statistically significant in the usual GEE model. Based on this table, higher number of transferred embryos is associated with an increase in the odds of success in a way that one unit increase in the number of transferred embryos is associated with 1.18 and 1.21-fold increase in the odds of success in unweighted and weighted GEE models, respectively. Having PCOS was associated with lower odds of success in IVF procedures but this association was not statistically significant in either models. Receiving frozen embryo transfers was associated with more than 2-fold increase in the odds of success in both models. 

**Fig.1:**
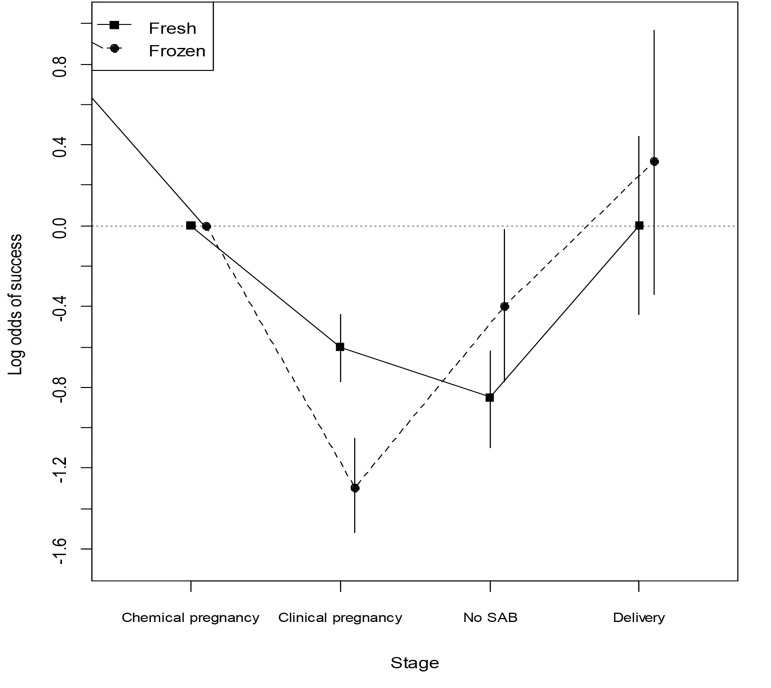
Log odds of success at multiple points during the IVF cycle with 95% confidence intervals. IVF; *In vitro* fertilization and SAB; Spontaneous abortion.

To explore the differing effect of fresh and frozen embryo transfer on the odds of success at various stages, the interaction term between type of embryo(s) transferred and failure type was included in the model. Although women receiving fresh embryo transfer showed significantly better results in clinical pregnancy, from then on, women receiving frozen embryo transfer could successfully continue in the same way as those receiving fresh embryos (Fig .1).

**Table 1:** Relationship between IVF outcomes and IVF/participants characteristics


IVF and participants characteristics	Unweighted GEE		Cluster weighted GEE	
	OR (95% CI)	P value	OR (95% CI)	P value

Intercepts				
Chemical pregnancy	1 ( reference)	-	1 ( reference)	-
Clinical pregnancy	2.11 (2.08, 2.15)	<0.001	2.12 (2.09, 2.18)	<0.001
SAB	2.20 (2.13, 2.29)	<0.001	2.22 (2.14, 2.34)	<0.001
Delivery	6.43 (3.42, 15.76)	0.010	8.69 (3.59, 30.03)	0.009
Embryos transferred number	1.18 (1.01, 1.38)	0.031	1.21 (1.03, 1.42)	0.021
PCOS				
Yes	0.74 (0.52, 1.06)	0.102	0.75 (0.52, 1.10)	0.138
No	1 ( reference)	-	1 ( reference)	-
Type of embryo(s) transferred				
Fresh	1 ( reference)	-	1 ( reference)	-
Frozen	2.50 (1.87, 3.35)	<0.001	2.26 (1.66, 3.07)	<0.001
Age categories (Y)				
<35	1 ( reference)	-	1 ( reference)	-
35-37	0.86 (0.38, 1.28)	0.460	0.87 (0.57, 1.31)	0.504
38-40	0.68 (0.46, 1.00)	0.052	0.67 (0.45, 1.00)	0.050
>40	0.74 (0.51, 1.07)	0.109	0.76 (0.52, 1.11)	0.161


IVF; *In vitro* fertilization, GEE; Generalized estimating equations, SAB; Spontaneous abortion, PCOS; Polycystic ovarian syndrome, CI; Confidence intervals, and OR; odds ratio.

## Discussion

There are some existing approaches to model IVF data including multiple cycles with multiple failure types (9). Considering the whole existing IVF data set for each woman can lead to better estimations of the covariates effects than the standard approach which only consider the first IVF cycle or model each IVF outcome separately.

Since the number of cycles experienced by each infertile woman is believed to be associated with the success/failure of IVF outcome, studies on these type of data involve informative cluster size and GEE and CWGEE, might show different results as GEE assumes that cluster size is non-informative. This historical cohort study on Iranian infertile women also demonstrated strong reverse associations between the number of cycles and odds of success in IVF outcomes, indicating the presence of informative cluster size (12). Moreover, the result of this study showed that having more transferred embryos is significantly associated with higher odds of success which corroborates the findings of previous research in this field (14, 15).

Based on both GEE and CWGEE, our results also suggest that successful IVF outcomes seem to be associated with performing frozen embryo transfer compared to fresh embryo transfer. This could be explained by the fact that the endometrium is more receptive in frozen embryo transfer during the endometrial priming than in fresh embryo cycles; therefore, frozen embryo cycles could lead to a better embryo-endometrium synchrony (16). Despite the potential advantages of transferring frozen embryos, the effect of patient-specific variables or center-specific factors (e.g. laboratory setup and protocols), should be investigated in well-designed clinical trials (17). Exploring the differing effect of frozen embryo transfer on the odds of success at various stages showed that the likelihood of successful clinical pregnancy is significantly lower in frozen embryo transferred cycles which could be explained by the fact that usually the best-quality embryos are chosen for the fresh embryo transfer and this is in agreement with previous studies (18, 19). Continuing through the cycles, the difference between frozen and fresh embryo transfer was not statistically significant which is probably due to the well-balanced embryo-endometrium interaction (16).

In our study, having PCOS was not significantly associated with odds of success in IVF procedures in either of the models which was not consistent with some previous research that found that women with PCOS have an increased prevalence of miscarriage, both after spontaneous and induced ovulation (10). However, this result is consistent with that of other studies which showed similar pregnancy and live birth rate per cycle in PCOS and non- PCOS women (20). Our limitation to include women’s BMI in this study could influence the results as the impact of BMI on IVF outcomes and its interaction with PCOS was not considered.

A great deal of previous research has indicated significant associations between women age and fertility (21, 22). In this study, although this association was not significant in GEE model, CWGG model confirms that being in the age category of 38-40 years old was reversely associated with odds of success compared to women aging less than 35 years old. The difference between the women aged under 35 years and those of over 40 years was not statistically significant which could be due to the limited number of women aged over 40 years old in our study.

In this study, data from repeated IVF cycles was used by including the correlation among them; however, not including some variables of couples undergoing IVF, such as pretreatment variables, embryo quality, oocyte and sperm quality and also stimulation and laboratory variables is a limitation of this study. Data on previous cycles, which infertile women might have undergone in other infertility centers, was not included in this study due to lack of a national registry.

## Conclusion

Frozen embryo transfer was positively associated with odds of success compared to cycles with fresh embryo transfer; but, cycles with fresh embryo transfer had better results in clinical pregnancy compared to frozen embryo transfer. The number of embryos transferred and women’s age were significantly associated with odds of success.
